# SOCIAL MEDIA–BASED BIBLIOTHERAPY FOR INFORMAL CAREGIVERS OF PEOPLE WITH DEMENTIA: A PILOT RCT

**DOI:** 10.1093/geroni/igae098.3139

**Published:** 2024-12-31

**Authors:** Shanshan Wang, Leung Sze Him Isaac, Fan Avis

**Affiliations:** The Hong Kong Polytechnic University, Hong Kong, Hong Kong, China (People’s Republic); The Chinese University of Hong Kong, Hong Kong, China (People’s Republic); The Chinese University of Hong Kong, Hong Kong, China (People’s Republic)

## Abstract

**Background:**

Informal caregivers of individuals with dementia are often overlooked as they themselves experience mental health issues. This randomized controlled trial aimed to assess the efficacy of social media-based bibliotherapy in improving the mental health of informal caregivers.

**Methods:**

A total of 60 informal caregivers were enrolled. Participants were randomly assigned to either the social media-based bibliotherapy group, which received eight weekly sessions of electronic bibliotherapy, or the usual care group, which only received routine services from community centers. Outcomes were assessed at baseline and immediately post-intervention. Descriptive statistics, t-tests, Mann-Whitney U tests, χ2 tests, and generalized estimating equations were used for quantitative data analysis.

**Results:**

The average age of caregivers was 57.41 (SD, 13.63), with a majority being female (79.3%). Baseline characteristics were similar between groups. The findings demonstrated that social media-based bibliotherapy was well received for informal caregivers, with WhatsApp being the preferred medium in this study. The efficacy on mental health (Wald χ2=8.918, p=.003) and all the subscales of stress (Wald χ2=4.198, p=.040), anxiety (Wald χ2=7.667, p=.006), depression (Wald χ2=9.127, p=.003) was statistically significant. The efficacy on caregiving appraisal was only significant on the perceived caregiving burden subscale (Wald χ2=4.954, p=.026). The efficacy on the mental component scale of health-related quality of life approached significance (Wald χ2=3.634, p=.057). Conclusions: The utilization of social media-based electronic bibliotherapy was well received among informal caregivers of individuals with dementia, demonstrating potential positive effects on their mental health within this specific cohort.

